# Gut Microbiota: A Novel Regulator of Cardiovascular Disease and Key Factor in the Therapeutic Effects of Flavonoids

**DOI:** 10.3389/fphar.2021.651926

**Published:** 2021-06-16

**Authors:** Qinyu Li, Bing Gao, Bateer Siqin, Qian He, Ru Zhang, Xiangxi Meng, Naiheng Zhang, Na Zhang, Minhui Li

**Affiliations:** ^1^ Department of Pharmacy, Baotou Medical College, Baotou, China; ^2^ Xilinguole Meng Mongolian General Hospital, Xilinhaote, China; ^3^ Pharmaceutical Laboratory, Inner Mongolia Institute of Traditional Chinese Medicine, Hohhot, China; ^4^ Inner Mongolia Key Laboratory of Characteristic Geoherbs Resources and Utilization, Baotou Medical College, Baotou, China; ^5^ Office of Academic Research, Qiqihar Medical University, Qiqihar, China

**Keywords:** gut microbiota, cardiovascular disease, interactions, metabolite, flavonoid

## Abstract

Cardiovascular disease is the main cause of death worldwide, and traditional cardiovascular risk factors cannot fully explain the occurrence of the disease. In recent years, the relationship between gut microbiota and its metabolites and cardiovascular disease has been a hot study topic. The changes in gut microbiota and its metabolites are related to the occurrence and development of atherosclerosis, myocardial infarction, heart failure, and hypertension. The mechanisms by which gut microbiota and its metabolites influence cardiovascular disease have been reported, although not comprehensively. Additionally, following ingestion, flavonoids are decomposed into phenolic acids that are more easily absorbed by the body after being processed by enzymes produced by intestinal microorganisms, which increases flavonoid bioavailability and activity, consequently affecting the onset of cardiovascular disease. However, flavonoids can also inhibit the growth of harmful microorganisms, promote the proliferation of beneficial microorganisms, and maintain the balance of gut microbiota. Hence, it is important to study the relationship between gut microbiota and flavonoids to elucidate the protective effects of flavonoids in cardiovascular diseases. This article will review the role and mechanism of gut microbiota and its metabolites in the occurrence and development of atherosclerosis, myocardial infarction, heart failure, and hypertension. It also discusses the potential value of flavonoids in the prevention and treatment of cardiovascular disease following their transformation through gut microbiota metabolism.

## Introduction

Cardiovascular disease (CVD) is the largest contributor to mortality and morbidity worldwide, accounting for 31% of all global deaths (Who, 2017). CVD is a manifestation of both a systemic circulatory disease and a problem localized to the heart. There are four main causes: 1) vascular factors, such as atherosclerosis (Stefanadis, 2013), hypertensive arteriosclerosis, and arteritis; 2) hemodynamic factors, such as hypertension (Sanchez-Rodriguez et al., 2020); 3) abnormal hemorheology, such as hyperlipidemia (Ma et al., 2019) and diabetes; and 4) blood composition factors, such as leukemia, anemia, and thrombocytosis. Presently, statins play a very important role in controlling CVD development by effectively controlling the blood lipid level, thus, alleviating the rapid progression of CVD. However, in some patients this effective control could not be observed even after the use of high-intensity statins. Additionally, statins have certain side effects, most commonly gastrointestinal reactions and possibly, individual allergic reactions to the different drugs that constitute statins. In serious cases, liver damage may occur. Hence, there is an urgent need to identify new effective targets for CVD and to investigate new therapeutic approaches.

Gut microbiota (GM) is a complex ecosystem of trillions of microorganisms (Qin et al., 2010; Man et al., 2020). Upon being affected by external environmental changes, the balance between the host and the flora can be broken, resulting in an imbalance in the intestinal micro-ecosystem, thus leading to impaired body functions and disease (Lau et al., 2017). With increasing developments in scientific research, the important role of GM in CVD development has been discovered. GM can improve CVD prognosis by reducing several risk factors, and its regulation is expected to become a new therapeutic target for CVDs. Close information exchange between GM and the host plays a vital role in digestion, immune defense, and nervous system regulation, especially cardiovascular protection, maintaining a delicate balance between itself and the human host (Guo and Wu, 2017; Jia et al., 2019b). Furthermore, it is important to study the relationship between GM and flavonoids for the protection of flavonoids to CVDs.

Flavonoids are the most common group of dietary polyphenolic compounds that are present in their glycosidic forms and found in a wide variety of foodstuffs of plant origin (Manach et al., 2004; Lu et al., 2017; Bi, 2018). They have a variety of biological activities (*in vitro* and *in vivo*) (Gabriele, 2015), including antibacterial, anti-inflammatory (Gabriele, 2015), antitumor, and antioxidant activities (Gabriele, 2015), as well as significant therapeutic effects on CVD (Manson, 2002; Bazzano et al., 2003; Gong et al., 2019). Flavonoids are recognized as xenobiotics and most glycosides are not easily transported to the target site because of their low lipophilicity, resulting in poor absorption and relatively low bioavailability (Karlsson et al., 2012; Kumar and Pandey, 2013; Man et al., 2020). Therefore, it is important to note that during GM growth and metabolism, GM can produce an array of metabolic enzymes, such as α-rhamnosidase, β-glucuronidase, β-glucosidase, β-galactosidase, nitroreductase, azoreductase, 7-α hydrolase, protease, and various carbohydrate metabolism-related enzymes (Gong et al., 2020). Flavonoids are metabolized by these GM enzymes and can be converted into their corresponding phenolic acids, thus affecting the bioavailability of flavonoids in the human body (Pan et al., 2013) and increasing the bioactivity of some flavonoid metabolites. On the other hand, the composition of GM can also be modulated by flavonoids toward homeostasis recovery (Akyildiz et al., 2010). Hence, it is important to study the relationship between GM and flavonoids to elucidate the protective effects of flavonoids in CVDs.

In this review, we comprehensively evaluate recently published scientific literature on the interaction between GM and flavonoids, with particular emphasis on the metabolized and transformed pathways involved. Further, we explain how flavonoids prevent CVDs through GM and discuss the roles of the GM in CVDs, including atherosclerosis, hypertension, myocardial ischemia (MI), and heart failure (HF), as well as the molecular mechanisms underlying its effects.

## Interaction Between Flavonoids and Gut Microbiota

Flavonoid metabolism by human GM and the effect of flavonoids on the growth of GM are mutually causal. On the one hand, when flavonoids enter the intestinal tract, they inevitably become metabolized and transformed through the action of various drug-metabolizing enzymes produced by the GM, leading to the formation of various bioactive metabolites, which have a positive effect in improving CVDs. On the other hand, flavonoids affect GM balance (Gil-Cardoso et al., 2016), which is closely related to human health. Thus, the interaction between flavonoids and GM is of great significance for intestinal health.

### Role of Gut Microbiota in Flavonoid Metabolism

**FIGURE 1:**
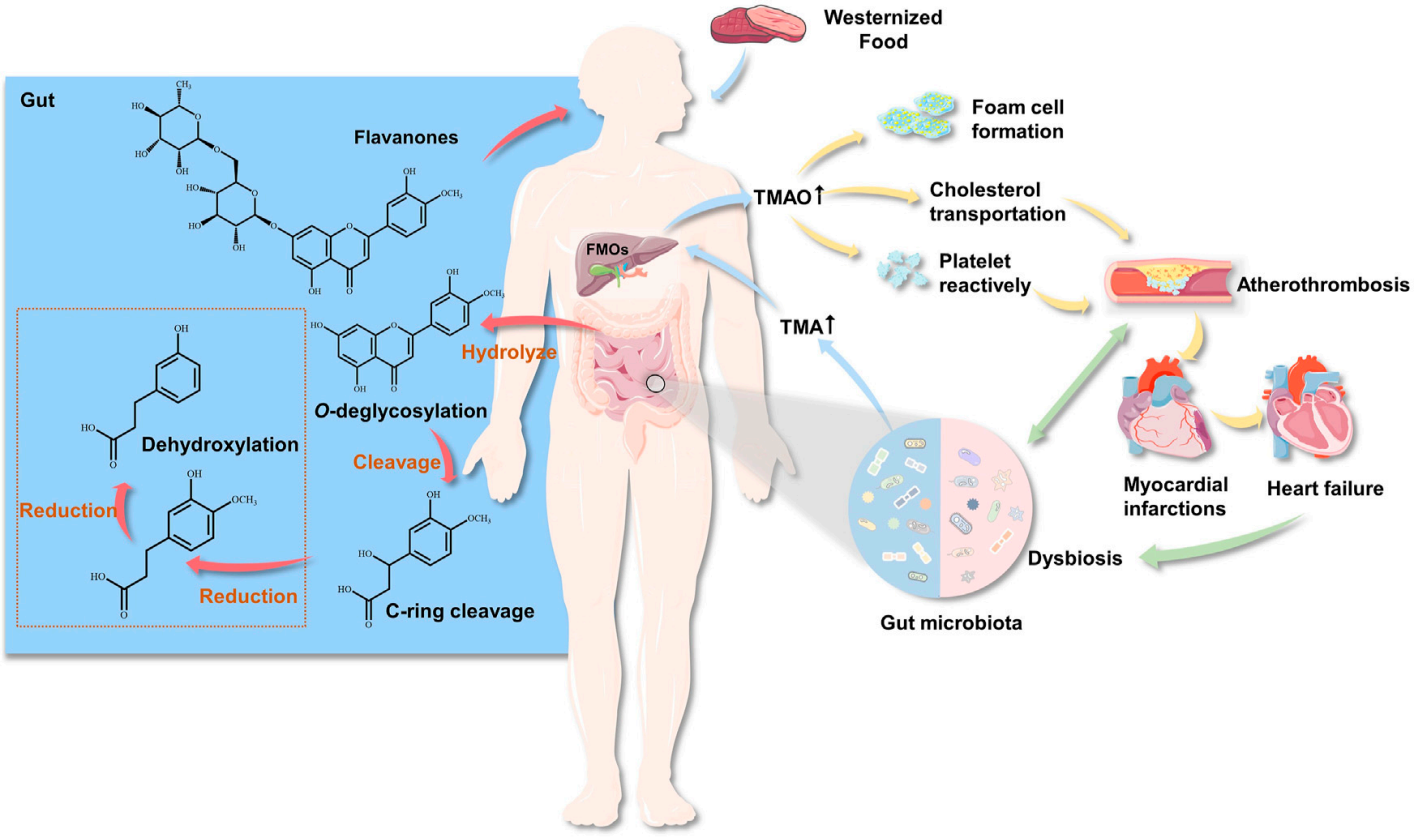
The role of gut microbiota in cardiovascular disease and flavonoid metabolism. The GM produces TMA by ingesting choline (such as choline, phosphatidylcholine and l–carnitine) from food. TMA enters the liver through the hepatointestinal circulation, and forms TMAO through the oxidation of flavin monooxygenase, then enters the systemic circulation. Circulating TMAO levels increase, causing foam cells and platelets to accumulate, while affecting cholesterol transport, thereby promoting atheromatous plaque formation.

*In vivo*, flavonoid metabolism is closely associated with GM. Microorganisms interact and cooperate with each other for the completion of complex metabolic functions. Under the action of GM, flavonoid glycosides are first hydrolyzed to aglycones in the intestinal tract, then reduced, with the hydrogenation of the C-ring, after which O–C2 bonds in the C-ring are cleaved to form phenolic ketones and phenolic acids (Figure 1). Therefore, hydrolysis and reduction are the main reactions of flavonoids in intestinal metabolism. In addition, GM can perform degradation of glycoside flavonoid macromolecules O-glycoside and C-glycoside, ester hydrolysis, hydrolysis (Jin et al., 2007), and deglucuronylation; partial dehydrogenation (Hor-Gil and Fatemeh, 2000), dimethoxylation (Shi et al., 2014), and demethylation of aglycones; and hydrogenation, α-oxidation, β-oxidation, and cracking of aromatic rings of substituted aliphatic groups. After the aromatic ring of the flavonoid aglycone is cracked, the hydroxylated forms of phloroglucinol and phenylacetate or phenylpropionate are produced.

For example, the main components of *Scutellaria baicalensis* Georgi include baicalin, wogonoside, and xyloside. These glycosides are converted by GM into the corresponding aglycones, such as baicalein, wogonin, and xyloside A, respectively, with a higher anti-complement activity than that of the prototypes. Antibacterial activity evaluation showed that the activities of all aglycones were stronger than those of the corresponding glycosides (Xing et al., 2014). With respect to anti-platelet aggregation, baicalein activity was significantly stronger than that of baicalin, which has a positive effect on improving atherosclerosis. It has been found that baicalin is first transformed into its aglycone baicalein in the intestinal tract of rats, absorbed, then converted into baicalin in the small intestine and liver (Akao et al., 2000). These findings suggest that the insoluble glycosides may be hydrolyzed into easily absorbed aglycones to make them better absorbed by the small intestine, and then converting them back to glycosides using host enzymes, thereby promoting glycoside absorption. Hesperidin, a flavanone glycoside, is a natural phenolic compound with a wide range of biological effects (Hajialyani et al., 2019). To clarify hesperidin metabolism by GM, hesperidin was incubated with human and rat fecal suspensions. By comparison, when hesperidin was orally administered to rats, hesperetin was detected in the urine, but hesperidin was not, indicating that hesperidin was metabolized to hesperetin, which was the main metabolite during incubation (Lee et al., 2004). Hesperidin can dilate blood vessels, and reduce blood lipid levels, capillary permeability, and vascular wall fragility. Protocatechuic acid is the main metabolite of anthocyanin fermented by human GM. It exhibits obvious antioxidant activity, scavenging of free radicals, inhibition of platelet aggregation, and anti-hypoxia activity, which are significant for improving CVDs. The appearance of low molecular weight metabolites indicates that anthocyanin has been transformed by GM (He et al., 2005). Kaempferitrin is a flavonoid glycoside isolated from *Siraitia grosvenorii* (Swingle) C. Jeffrey ex Lu and Zhang, which is incubated and transformed by the bacteria in human intestines to produce afzelin, kaempferol, p-hydroxybenzoic acid, and kaempferol-7-O-α-L-rhamnoside (Yang et al., 2005). It has various pharmacological activities, such as lowering of blood pressure, reduction of blood lipid levels, and inhibition of platelet aggregation.

### Effect of Flavonoids on Gut Microbiota

Flavonoids can promote the growth of beneficial intestinal microorganisms. Flavonoids, such as hesperidin and quercetin have been reported to modulate the composition of GM by promoting the growth of probiotic bacteria and effecting a reduction in the growth of pathogens. A study conducted by Simmering (Rainer et al., 2002) examined the influence of flavonoids on the population of *Eubacterium ramulus* in the human intestinal tract. A controlled study was performed with human subjects who received a flavonoid-free diet and a single high dose of quercetin, rutin, or buckwheat. Results showed that oral quercetin and rutin intake significantly increased the bacterial load of *E. ramulus* in humans. Metabolomic analyses have also shown that grape seed extract, rich in flavanols, had a strong value-added effect on the proliferation of *Lactobacillus plantarum*, *Lactobacillus casei*, and *Lactobacillus bulgaricus* (Tabasco et al., 2011).

Moreover, flavonoids have anti-microbial activity, and a large number of studies have shown that they can inhibit the proliferation of symbiotic bacteria and pathogenic microorganisms, and thus have a certain impact on CVD. The quantity of *Lactobacillus* and *Escherichia coli* per gram of fecal contents in rats fed soybean isoflavones was calculated using the bacterial culture method. The results showed that the erobic *E. coli* numbers in the intestinal tract of rats fed soybean isoflavone decreased significantly, while the count of the facultative anaerobic *Lactobacillus* spp. increased significantly (Zhu et al., 2013). This is similar to the results of a study by Duda-Chodak (Duda-Chodak, 2012) in which the liquid medium was supplemented with various polyphenol monomers, whose effects on GM were evaluated by measuring the turbidity of the medium after 24 h. It was found that quercetin significantly inhibited the growth of *Bacteroides galactosus* and *Lactobacillus*. Furthermore, Gwiazdowska (Daniela et al., 2015) studied the effects of different polyphenol monomers (2, 20, and 100 μg/ml) on the growth of *Bifidobacterium Bifidum* using the 96-well microtitration plate method. The results showed that rutin and quercetin promoted the growth of *B. Bifidum*, but high-dose quercetin inhibited the growth of *B. Bifidum*. These observations indicate that the effect of flavonoids on GM depends on the dosage of flavonoids, and different concentrations of flavonoids have different inhibitory effects on different bacterial strains. Currently, flavonoids, as antibacterial drugs, have been widely used in the medical and food preservative industries.

## Flavonoids Prevent Cardiovascular Disease Through Gut Microbiota

Flavonoid intake is closely associated with CVD. The interaction between flavonoid glycosides and GM can produce a new type of microbial flavonoid transformation with significantly increased biological activity and bioavailability, thus promoting the full use of flavonoids in cardiovascular protection (Table 1).

**TABLE 1:** The interaction of flavonoids and gut microbiota.

Class	Compounds	Dietary source	Gut microbiota/Enterobacterial metabolic enzyme	End-products	End-products biological function	Ref.
Flavonol	Quercetin	Onions, kale, broccoli, beans, black currants, apples, tea	*Clostridium orbiscindens*, *Eubacterium oxidoreducens, Butyrivibrio* spp.	Phenylacetic acid, 4-HPPA, protocatechuic acid, (3,4-dihydroxy phenyl) propionic acid, and 4-hydroxybenzoic acid	Antitumour, and anti-inflammatory	Winter et al. (1989), Hollman and Arts. (2000); Bowey et al. (2003), Braune and Blaut. (2011), Luo et al. (2014)
	Kaempferol	Fruits, vegetables, and tea	*Clostridium* strains	Phenylacetic acid	Antioxidation	Simpson et al. (1960), Winter et al. (1989), Guo et al. (2014), Villarreal et al. (2019)
	Icariin		*Streptococcusmrg*-ICA-*benterococcusmrg*-ICA-E, and *Blautia* MRG-PMF-1	Icariside II, icartin, and desmethylicaritin	Antitumour, and immunoregulation	Wu et al. (2016a); He et al. (2020)
Flavanones	Hesperidin	Citrus fruits	Human fecal flora	Hesperetin	Anti-inflammatory, antioxidation, antibacterial, and anticancer cardiovascular	Kim et al. (1998), Tomás-Barberán and Clifford. (2000)
	Naringenin	Citrus fruits	*Clostridium* strains	3-(4-hydroxyphenyl) propionic acid, phlorogucinol	Free radical scavenging, anti-hyperlipidemic	Winter et al. (1989), Tomás-Barberán and Clifford. (2000)
Flavones	Luteolin	Sweet red pepper, cellery, cauliflower	Rat fecal flora	3-(2,4-dihydroxyphenyl)- propionic acid	Antitumour, anti-inflammatory, and antioxidation	Hollman and Arts. (2000), Aura. (2008), Wang et al. (2013)
	Baicalin		β-D-glucuronidase	Baicalein	Free radical scavenging, anti-tumour, and anti-HIV	Chang et al. (2008), Assini et al. (2013)
	Apiin	Sweet red pepper, cellery, cauliflower	*Bacteroides distasonis* and *Eubacterium ramulus*	Apigenin, naringenin, and p-hydroxyphenyl propionic acid	Antidepressant	Hollman and Arts. (2000); Liu and Zhao. (2004); Serra et al. (2012)
Isoflavonoids	Daidzein	Soy products	*Bacteroide ovatus* spp., *Streptococcus intermedius* spp., *Ruminococcus productus*, SNU-Julong 732 (AY310748), *Enterococcus faecium* EPI1, *Lactobacillus mucosae* EPI2, *Finegoldia magna* EPI3, and *Veillonella* spp. EP	Equol	Anti-diabetic and myocarditis	Hur et al. (2002), Lilian et al. (2002), Hanske et al. (2009), Cassidy et al. (2010), Yin et al. (2016)
	Genistein	Soy products	-	Dihydrogenistein, 6′-Hydroxy-*O*-demethylangolensin	Anti-bacteria	Cassidy et al. (2010)
Flavan-3-ol	Catechin and Epicatechin	Tea, red wine, chocolate	*Clostridium cocoides*-*Eubacterium rectale* group	3-(3-hydroxyphenyl) propionic acid, 5- (30,40-dihydroxyphenyl)-γ-valerolactone, 5-(30-hydroxyphenyl)-γ-valerolactone, 3-hydroxyhippuric acid pyrogallol, 5-(3,4-dihydroxyphenyl) valeric acid	Anti-oxidation, anti-bacteria and protect the heart and brain organs	Hollman and Arts. (2000), Tzounis et al. (2008)

On the one hand, because of their low lipophilicity, most flavonoid glycosides are difficult to transport to the target site, resulting in poor absorption of flavonoid glycosides in the digestive tract. However, after biotransformation of some flavonoids by GM, the flavonoid metabolites produced show a greater biological activity than the parent flavonoid. For example, a large number of epidemiological studies have shown that soybean isoflavones have many physiological functions, such as anti-cancer, anti-oxidation, and anti-inflammatory properties, cardiovascular and cerebrovascular protection, and osteoporosis prevention. Soybean isoflavones ingested by humans and other mammals can be degraded into dihydrodaidzein, dihydrogenistein, O-desmethylangolensin, estriol, and other metabolites by GM. *In vivo* and *in vitro*, the results showed that soybean isoflavone metabolites had higher and wider biological activity than soybean isoflavone. Besides, estriol has a higher affinity for thromboxane receptors in human platelets than soybean isoflavones; therefore, it can be used clinically to regulate platelet function and prevent thrombus. Compared with isoflavones, the conformation of estriol is more flexible, allowing its easier penetration into the cell membrane. Its antioxidant activity plays an important role in preventing lipid peroxidation and has positive effects on the treatment and prevention of chronic diseases, preventing atherosclerosis, and reducing the risk of CVD.

On the other hand, there are substantial differences in the efficacies of some flavonoids *in vitro* and *in vivo*. One of the important reasons is the low solubility or poor absorption *in vivo*, which leads to poor bioavailability. The metabolism of flavonoids by GM can improve their water solubility and bioavailability. For example, rutin is a flavonoid obtained from a wide range of sources. It can prevent and treat hemorrhage caused by capillary embrittlement. It is used clinically in the auxiliary treatment of hypertension and it performs anti-capillary fragility activity. However, rutin is poorly soluble in water and fat, with low bioavailability after oral administration, which limits its clinical application. Rutin can be hydrolyzed to quercetin by GM and finally converted to 3,4-dihydroxyphenylacetic acid, the antiplatelet activity of which is greater than that of rutin and quercetin, and also it can be absorbed into the blood circulation system. Consequently, in the preparation of rutin as the main effective ingredient, in addition to the dosage form, we can also consider increasing the residence time of rutin in the intestinal tract, in order to enhance its metabolism by GM and its cardiovascular protection efficacy.

## Mechanisms Underlying the Improvement of Cardiovascular Diseases by Gut Microbiota

**FIGURE 2:**
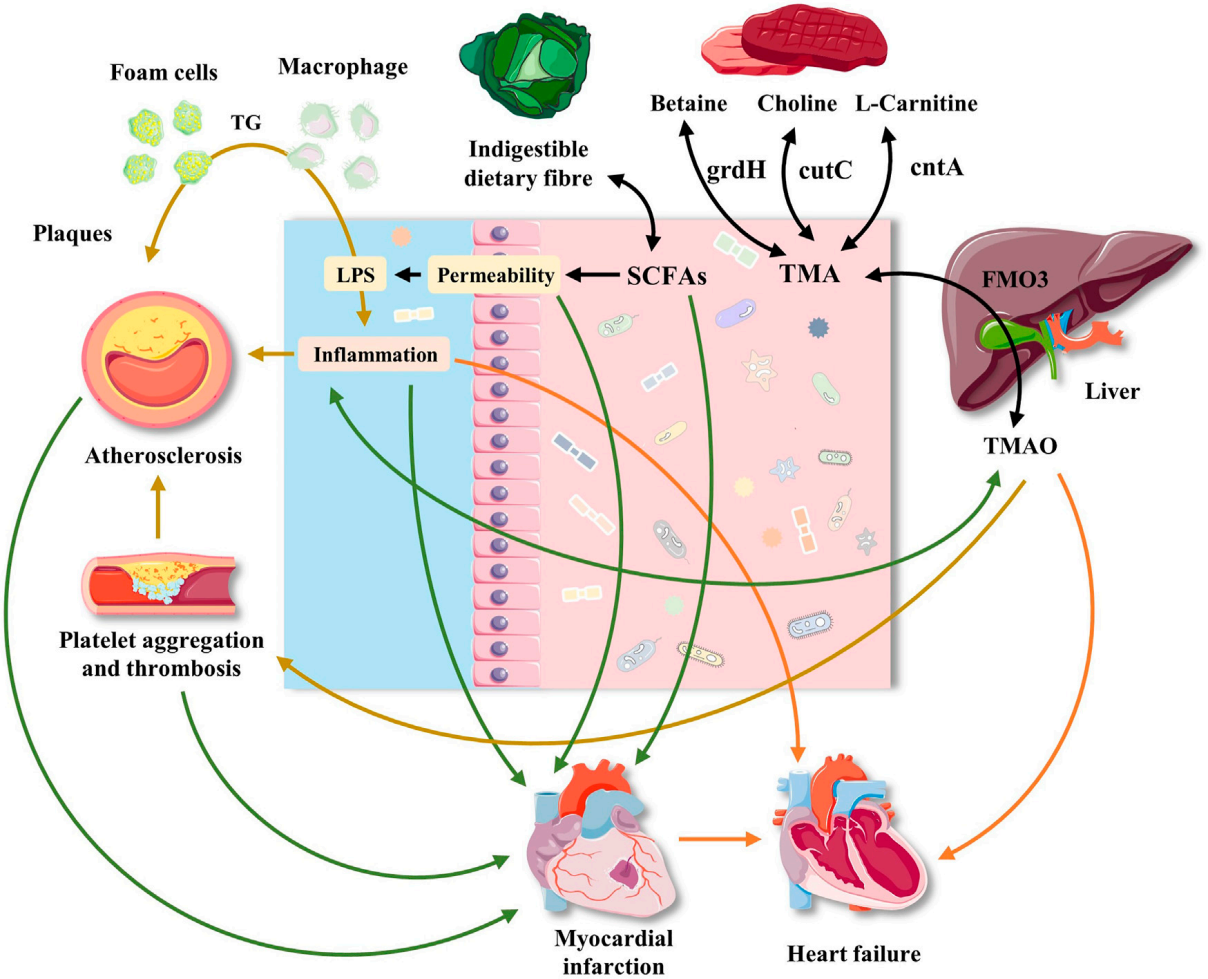
Effects of gut microbiota and its metabolites on atherosclerosis, myocardial infarction, heart failure and hypertension. The interaction between diet and gut microbiota may promote the development of cardiovascular disease through common and different mechanisms. Western food rich in red meat promotes the production of TMA by bacteria, which is oxidized to TMAO in liver. TMAO may participate in atherosclerosis by interfering with cholesterol transport, foam cell formation and platelet aggregation. The effect of TMAO on blood pressure was mainly manifested in its enhancement of pressure-raizing effect of Ang II. Platelet aggregation also plays a role in atherosclerosis. The decrease of dietary fiber is related to the decrease of bacterial production of SCFAs, which play an immunomodulatory role in intestinal mucosa. The decrease of SCFAs level can promote local inflammation, aggravate intestinal ecological imbalance, and lead to the damage of intestinal barrier function. The damage of intestinal barrier function leads to the leakage of bacterial toxins, which further aggravates local and systemic inflammation. In addition, dysbiosis destroys the intestinal mucosal barrier, leading to gut microbiota entering the systemic circulation, which increases the incidence of adverse cardiovascular events after MI and aggravates the progress of HF. The roles of gut microbiota and its metabolites in hypertension.

Trimethylamine N-oxide (TMAO) is formed from carnitine-, choline-, and betaine-rich foods, such as egg yolk, red meat, and some fish, through the action of trimethylamine (TMA) lyase in intestinal microorganisms, followed by oxidation by liver flavin monooxygenases (FMOs; mainly FMO3). Table 2 lists the microorganisms involved in metabolizing carnitine, choline, and betaine (Aura, 2008). Additionally, short-chain fatty acids (SCFAs) are organic acids with aliphatic terminal carboxylic acids, which are mainly produced by anaerobic bacteria in the cecum and proximal colon through the fermentation of dietary fiber, with a small portion produced by the decomposition of proteins and peptides (Pingitore et al., 2017). SCFAs play a regulatory role in energy homeostasis, insulin sensitivity, and glucose and lipid metabolism. It is generally accepted that these regulatory roles of SCFAs can be used to reduce the risk of cardiovascular and metabolic diseases (Donohoe et al., 2018). Biologically active GM-derived metabolites, such as TMAO and SCFAs, are bioactive molecules and a promoter of chronic CVDs (Romano et al., 2015; Roberts et al., 2018). Moreover, alterations in the human gut-associated microbiome have been implicated in the pathogenesis of several chronic conditions ranging from atherosclerosis and thrombosis to hypertension and HF (Tang and Hazen, 2017) (Figure 1). The following sections will elaborate on the effect of GM and its metabolites on atherosclerosis, MI, HF, hypertension, and related mechanisms (Figure 2).

**TABLE 2:** The microorganisms affecting the metabolism of TMAO, carnitine, choline and betaine.

Classification	Genus or species	References
TMAO		
* Actinobacteria*	*Micrococcus*, *Mobiluncus*	Robinson et al. (1952), Cruden and Galask. (1988)
*Firmicutes*	*Bacillus*, *Clostridium*, *Staphylococcus*, *Sarcina*	Robinson et al. (1952)
*Proteobacteria*	*Campylobacter*, *Escherichia*, *Pseudomonas*	Sellars et al. (2002), Chen et al. (2011), Denby et al. (2015)
Choline		
*Actinobacteria*	*Olsenella*, *Mobiluncus Spiegel and Roberts*	Craciun and Balskus. (2012), Martínez-del Campo et al. (2015), Fennema et al. (2016)
*Firmicutes*	*Anaerococcus*, *Clostridium*, *Streptococcus*, *Desulfitobacterium*, *Bacillus*	Craciun and Balskus. (2012), Romano et al. (2015); Martínez-del Campo et al. (2015), Fennema et al. (2016), Rath et al. (2017)
*Proteobacteria*	*Desulfovibrio*, *Edwardsiella*, *Proteus*, *Providencia*, *Yokenella*	Craciun and Balskus. (2012), Romano et al. (2015), Fennema et al. (2016)
Carnitine		
*Proteobacteria*	*Acinetobacter*, *Citrobacter*, *Escherichia*, *Klebsiella*, *Bacillus*	Zhu et al. (2014), Meadows and Wargo. (2015), Rath et al. (2017)
Betaine		
*Firmicutes*	*Eubacterium*, *Sporomusa*, *Fusiformis*	Hormann and Andreesen. (1989), Fennema et al. (2016)

Hypertension is one of the most common CVDs and its incidence rate increases with age. Its pathogenesis is complex and has many influencing factors (Li et al., 2017). More recently, a limited number of studies have indicated a direct association between GM and blood pressure control in animal models (Yang et al., 2015). GM produces unique metabolites that are potentially important for hypertension control. It was found that the diversity, abundance, and uniformity of GM decreased in the animal model of spontaneous hypertension, while the ratio of *Firmicutes* to *Bacteroidetes* increased (Yang et al., 2015; Karbach et al., 2016). In one study, the GM of 41 healthy volunteers, 56 patients with prehypertension, and 99 patients with essential hypertension were analyzed. Additionally, the researchers transplanted the fecal microbiota from the patient into the intestines of sterile mice and observed a significant increase in mice blood pressure after transplantation (Li et al., 2017).

In recent years, SCFAs have been shown to act on G-protein-coupled receptors (GPCRs), including free fatty acid receptor 3 (Gpr41) and olfactory receptor 78 (Olfr78), to regulate blood pressure. Short-chain fatty acids (SCFA) activate three independent GPCRs, namely GPR41, free fatty acid receptor 2 (GPR43), and Olfr78. SCFA can activate Olfr78, which makes renin released from renal arterioles increase hypertension, (Pluznick, 2017), while GPR41 shows an antagonistic effect (Natarajan et al., 2016). In contrast, SCFA activates GPR43 to induce vasodilatation to counteract increased blood pressure induced by Olfr78-mediated renin release (Chiou and Burotto, 2015). However, it has also been suggested that GPR41 and vascular Olfr78 have opposite effects on blood pressure regulation after propionate reaction (Pluznick et al., 2013; Jia et al., 2019a). In addition, compared with the traditional culture mice, the administration of angiotensin II in aseptic mice can reduce the formation of vasoactive oxygen species, decrease the levels of monocyte chemoattractant protein, and reduce the adhesion of vascular leukocytes, neutrophils, and monocytes to the vascular wall, thereby reducing vascular endothelial dysfunction and increasing blood pressure.

Further, the 3-years incidence of cardiovascular events is significantly higher in patients with high plasma TMAO concentration (Tang et al., 2013), which might be related to the increased degree of atherosclerosis associated with TMAO. Animal experiments identified elevated TMAO levels in mice accompanied by significantly increased areas of plaque and a higher number of foam cells (Wang et al., 2011; Koeth et al., 2015). However, the effect of TMAO on blood pressure mainly manifests as an enhanced pressor effect of angiotensin II (Ang II). Although simple infusion of Ang II only significantly increases the systolic and diastolic blood pressure of rats, the pressor effect can last until the end of the experiment when TMAO is injected simultaneously. Similarly, TMAO alone had no effect on the blood pressure of rats, whereas its combination with low-dose Ang II prolonged the Ang II-mediated pressor effect (Ufnal et al., 2014). A possible mechanism might involve TMAO participation in the protein processing of some Ang II receptors or Ang II-like peptide hormones, which improves the efficiency of protein production and thereby prolongs the Ang II-mediated pressor effect (Wu et al., 2016b).

### The Roles of Gut Microbiota and its Metabolites in Atherosclerosis

Atherosclerosis, one of the main diseases that harm human cardiovascular and cerebrovascular health is mainly caused by lipid metabolism disorder and vascular endothelial inflammation (Chistiakov et al., 2015). It is a chronic inflammatory reaction of the arterial wall (Wu M. Y. et al., 2017), mainly manifested by the formation of atherosclerotic plaques on the arterial wall, resulting in loss of arterial elasticity and eventually leading to adverse cardiovascular events (Greaves and Gordon, 2013; Mailer et al., 2017; Peter, 2017). Recent studies show that the changes in GM composition and diversity are closely related to the occurrence of chronic low-grade inflammation and atherosclerosis (Jones et al., 2013; Witjes et al., 2015); it was observed that the *Yersinia* and *Rhodobacter* population decreased, while that of *Collinsella erofaciens* and monocytes increased (Svetel et al., 2013). This was illustrated in a study of the feces of 12 patients with obvious symptoms of carotid atherosclerotic plaques and 12 healthy individuals matched according to gender and age (Karlsson et al., 2012). The data showed that the number of coliforms in the intestinal tract of the patients increased, while the number of *Rhodobacter* and *Eubacterium* increased in the healthy controls. Further, in a metagenomic association study on feces from 218 individuals with atherosclerotic CVD and 187 healthy controls (Jie et al., 2017), significant differences in GM were found between individuals with atherosclerotic CVD and the control group, including a relative reduction in *Bacteroides* and *Prevotella* populations, and the enrichment of *Streptococcus* and *E. coli*.

GM can affect the development of atherosclerosis through a variety of ways. It can not only promote an inflammatory reaction, aggravate the development of atherosclerotic plaque, or lead to plaque rupture but also affect the occurrence and development of atherosclerotic plaque by regulating host cholesterol and lipid metabolism. It is generally believed that GM is an activator of inflammation, especially that related to lipopolysaccharide (LPS) synthesized by Gram-negative bacteria and a strong inflammatory trigger (Du et al., 2017). LPS produced by GM can promote the progression of chronic low-grade inflammation, which is recognized as a risk factor for atherosclerosis. GM dysbiosis is accompanied by increased intestinal permeability, which leads to increase in endotoxin levels in the blood. Additionally, high-dose LPS can significantly increase the expression of nuclear factor kappaB (NF-κB) and other inflammatory factors, whereas low-dose LPS can induce macrophage differentiation into a mild proinflammatory state through Toll-like receptor 4, interleukin-1 receptor-related kinase-1, and Toll-interacting protein on the cell surface and promote the occurrence of atherosclerosis (Maitra et al., 2012). Moreover, GM inhibits the expression of fasting-induced adipocyte factor, increases triglycerides in blood circulation, and promotes deposition of adipocytes, resulting in increased lipid levels and leading to dyslipidemia and induction of atherosclerosis (Backhed et al., 2004). Furthermore, the signaling molecules produced by GM significantly influence cholesterol reverse transport. For example, low-dose endotoxin LPS can significantly reduce the expression of ATP-binding cassette (ABC) subfamily A member 1 and ABC subfamily G member 1 by cholesterol-reversion factor and inhibit the reverse process of cholesterol accumulation in macrophages, which promotes foam-cell accumulation in the vascular endothelium to form plaques and the occurrence of arteriosclerosis (Maitra and Li, 2013).

As a potential promoter of atherosclerosis and cardiac metabolic diseases, TMAO significantly affects the development of atherosclerotic lesions, resulting in increased attention being given to its molecular mechanisms in the human body (Tang et al., 2013). TMAO can not only significantly accelerate the development of atherosclerosis in apolipoprotein E-deficient mice but also activate the mitogen-activated protein kinase and NF-κB signaling pathways in endothelial cells and smooth muscle cells, thereby triggering inflammatory reactions in vessels and causing increased accumulation of foam cells in the vascular wall and atherosclerosis (Zeisel and Warrier, 2017). These findings suggest that reducing *in vivo* TMAO levels by inhibiting the proliferation of TMA-producing bacteria might improve the symptoms of atherosclerosis. One study reported that TMAO can inhibit the reverse transport of cholesterol and regulate the activity of cholesterol transporters in macrophages, thus leading to atherosclerosis (Koeth et al., 2015). Conversely, another study aimed at the key TMA enzyme (cutC/D) produced by bacteria and developed two strong inhibitors that can permanently inactivate cutC/D without affecting the survival ability of the symbiotic bacteria. After oral administration, the selective accumulation of the inhibitor in the intestinal symbiotic bacteria can almost completely inhibit the production of TMA and TMAO caused by hypercholinergic food, reduce platelet aggregation and thrombosis, and thus reduce the risk of atherosclerosis. Simultaneously, it reverses the changes in the intestinal bacterial composition induced by high choline foods, and increases the levels of *Akkermansia muciniphila* (Roberts et al., 2018). The TMA/TMAO pathway likely represents only one of many microbe-dependent pathways that will ultimately be linked to CVD pathogenesis. Early intervention of GM and its metabolites can help to better control the occurrence and progression of atherosclerosis, stabilize atherosclerotic plaques, and reduce the risk of MI.

### The Roles of Gut Microbiota and its Metabolites in Myocardial Ischemia

MI, caused by the necrosis of myocardium due to prolonged ischemia, was one of the most critical stress to the patients (Wu Z. X. et al., 2017). Nevertheless, different from other chronic CVDs, MI is a kind of severe stress injury. Under this kind of trauma, the structure of GM and its metabolites undergo stress changes. Patients with MI will have a typical GM dysbiosis, with a significant reduction of probiotics, a large number of pathogens, impaired intestinal barrier function, increased intestinal permeability and a series of changes. Based on the analysis of the blood microflora of 49 healthy controls, 50 patients with coronary heart disease, and 100 patients with MI, it was found that the diversity and richness of the blood microflora in patients with MI increased, and more than 12% of the bacteria in the blood was derived from GM (Zhou et al., 2018). Studies based on MI rat models have shown that the GM abundance was significantly higher in rats with MI than in the sham operation group, upon comparing the intestinal barrier injury of both rat groups (Wu Z. X. et al., 2017). Further, MI led to a decrease in *Lactobacillus*, and SCFAs were found to alleviate MI, indicating that GM and its metabolites have a significant repair effect on the cardiac tissue after MI. Similar studies have confirmed that the dysbacteriosis destroys the intestinal mucosal barrier, causing GM to enter systemic circulation, thus increasing the incidence of adverse cardiovascular events after MI. The incidence of adverse cardiovascular events increased significantly in patients with MI who had significantly increased GM (*Lactobacillus*, *Bacteroides*, and *Streptococcus*) populations (Org et al., 2015). Another study also found that the use of antibiotics to reduce the bacterial translocation in mice after MI can reduce the systemic inflammatory response and myocardial damage (Zhou et al., 2018).

Additionally, GM and its metabolites can, not only decrease the risk of MI by affecting intestinal barrier function, but also influence MI occurrence and development in other ways. Studies have shown that bacterial DNA found in arterial plaques is similar to that found in GM, and these bacteria may be related to plaque stability, implying that unstable atherosclerotic plaques increase the risk of MI (Koren et al., 2011). Elevated TMAO levels can affect the repair of mitochondria and myocardial metabolism and increase the risk and severity of MI (Makrecka-Kuka et al., 2017). TMAO can also promote the release of calcium ions into the cells through adenosine diphosphate, thrombin, and collagen, improve the sensitivity of platelets, and then promote thrombosis, which may directly stimulate the occurrence of MI (Zhu et al., 2016). Further experiments showed that antibiotics can reduce the MI area of acute myocardial infarction in mice, with the mechanism possibly related to the regulation of intestinal microorganisms (Lam et al., 2016). Furthermore, a recent study found that before myocardial infarction, probiotics supplementation in mice treated with antibiotics was beneficial at restoring cardiac function and prolonging survival time. That study found that GM-derived SCFAs played an important role in maintaining host immune components and repair ability after myocardial infarction, suggesting that regulation of intestinal flora might provide opportunities for rehabilitation after myocardial infarction (Tang et al., 2019); however, their ability to prevent coronary atherosclerosis and reduce the incidence of acute MI remains unclear. Thus, the mechanism by which GM and its metabolites influence the occurrence and development of MI is extremely complex, and needs to be studied further.

### The Roles of Gut Microbiota and Its Metabolites in Heart Failure

HF is the ultimate stage after MI. The structure and function of the heart are seriously damaged. Currently, surgery and drug treatment can delay the progress of HF to some extent, but the mortality rate is still very high. In recent years, great advances have taken place in understanding the pathophysiological mechanism of HF, from hemodynamics to neurohumoral immune regulation. The role of GM in HF has attracted widespread attention. In patients with HF, due to volume overload, intestinal congestion and ischemia could occur, leading to the dysbiosis of GM and an increase in intestinal permeability (Pasini et al., 2015). This will allow too many toxins to enter into blood circulation, activate cytokines, generate a systemic inflammatory response, and aggravate the progress of HF (Krack et al., 2004; Cannon and Mcmurray, 2014; Cui et al., 2018). Similar results were observed in a more recent study of patients with HF who were more likely to be infected with pathogenic bacteria than those in the control group (Pasini et al., 2015). *Candida*, *Campylobacter*, and *Shigella* were positively correlated with disease severity. Compared with the control group, 78.3% of the patients with HF had increased intestinal permeability, which was higher in patients with moderate and severe HF than in patients with mild HF. These results show that HF destroys GM balance, and the destruction of the intestinal mucosal barrier leads to bacterial translocation, an increase in systemic endotoxins, potential inflammation, and aggravation of HF.

Additionally, GM metabolites are closely related to HF. Recent studies have shown that TMAO is directly involved in the development of HF. TMAO levels were significantly higher in the blood of patients with HF than in that of healthy controls, and the prognosis of patients with high TMAO levels was poor (Tang et al., 2014). For instance, results from a study of a large number of chronic HF patients undergoing treatment show that increased TMAO content is positively correlated with the probability of death, worsening of patients’ condition, and probability of relapse in recovered patients within a short period of time (Chun and Rui, 2017). Further, Organ et al. (Organ et al., 2016) established a mouse HF model by surgery and found that mice fed TMAO showed more severe symptoms and signs of HF than mice fed a normal diet. Because 3,3-dimethyl-1-butanol can reduce the level of plasma TMAO, another animal study found that feeding 3,3-dimethyl-1-butanol to mice with HF induced by aortic coarctation significantly improved cardiac structural remodeling and electrical remodeling, suggesting a relationship between low levels of plasma TMAO and these improvements (Wang et al., 2020). Moreover, TMAO directly leads to progressive tubulointerstitial fibrosis and dysfunction, which might represent a potential mechanism associated with HF progression (Tang et al., 2015). Although there are several studies on the mechanisms underlying the effects of metabolites on HF, the GM composition and the specific mechanism by which the metabolites affect HF still need to be further studied.

## Conclusion and Future Prospects

In recent years, interest in studying the interaction between flavonoids and GM has increased. When flavonoids enter the intestinal tract, they inevitably become metabolized and transformed under the action of various drug-metabolizing enzymes produced by the GM, and produce bioactive metabolites, which can promote cardiovascular health by exerting systemic and local effects. A large number of microorganisms in the intestine can metabolize flavonoids that cannot be metabolized by the gastrointestinal tract. The metabolic products are absorbed by the body or help in resisting CVDs and exhibit certain biological activities. Meanwhile, flavonoids from different sources are beneficial for GM and also affect GM balance. They interact with GM, potentially inhibiting the growth of harmful microorganisms, promoting the proliferation of beneficial microorganisms, and maintaining GM balance. Therefore, GM, as an integral part of the human body, actively participates in the biotransformation process of flavonoids, thereby influencing the cardiovascular system. Further, GM metabolites also affect CVD.

Although the research on the relationship between GM and its metabolites and atherosclerosis, MI, HF and hypertension is growing rapidly. Further researches, however, are encouraged to focus on the host-microbiota interactions and related effects during the treatment of flavonoids on CVD. Potential challenges and problems in the future can be summarized as follows: 1) Limited development and application of flavonoids due to the lack of specificity and selectivity for CVDs because of their complex structure and multiple targets. 2) The difficulty in excluding individual GM differences, such as age, environment, diet, and other influencing factors. 3) Most of the data were obtained from rodents. Due to the differences in intestinal and body functions between rodents and humans, many research results cannot be directly applied to humans. 4) The effects of bacterial imbalance, intestinal barrier damage, and chronic inflammation associated with GM translocation, TMAO, SCFAs, etc., on CVD have been confirmed, but the mechanisms of action remain to be further studied.

In summary, this review shows that the intake of flavonoids could increase the proliferation of certain gut bacteria that attenuate CVDs, and the relationships among flavonoids, GM, and CVD could offer a new perspective to understanding the mechanisms underlying the effects of flavonoids on CVD. It should be noted that research on the absorption, distribution, and metabolism of flavonoids in the intestinal tract is still at the initial stage globally. After the metabolic transformation of flavonoids in GM, their effects on CVDs are unstable and the mechanism of action is unclear, which is one of the difficulties in its development and utilization. Therefore, it is necessary to further enrich and strengthen research on the *in vivo* analysis of the action mechanism of flavonoids, which is of great significance for guiding rational diets and improving the use of functional foods.
